# Effect of preservative and preservation methods on physical, chemical and microbiological properties of nipa palm (*Nypa fructicans*) sap

**DOI:** 10.1186/s13104-024-07039-5

**Published:** 2025-03-31

**Authors:** Dayang Nur Athirah, Mohd Razip Asaruddin, Showkat Ahmad Bhawani, Aldrin Felix Simbas, Kho Swen Jack

**Affiliations:** Universiti Malaysia Sarawak, Samarahan, Sarawak, Malaysia

**Keywords:** Food preservatives, Timing of preservative addition, Nipa palm sap

## Abstract

**Objectives:**

This study aimed to evaluate the effectiveness of the E’ food grade preservative – potassium sorbate (E202) and sodium metabisulfite (E223) in an aqueous form, and impact of the timing of preservative addition on the shelf-life, sugar and reducing sugars levels, organic acids content and microbial growth of nipa palm sap.

**Results:**

The pH, sugar and reducing sugars levels, organic acids content, total bacterial count (TBC) and sensory characteristics of the sample were determined. The results showed that the treated samples were able to slow down the pH and sucrose content from dropping during storage. Sample A had lactic acid at 0.93 ± 0.01 g/L and acetic acid at 0.05 ± 0.0.1 g/L, while Sample B had 0.97 ± 0.02 g/L and 0.07 ± 0.01 g/L, respectively, both lower than the control (1.10 ± 0.01 g/L and 0.09 ± 0.02 g/L). Sample A with TBC of 1.03 (± 0.08) x 10^5^ CFU/mL was able to inhibit microbial growth better than Sample B, 1.14 (± 0.13) x 10^5^ CFU/mL. Preservative application prior to tapping was shown to be more successful in preserving the quality and shelf-life of nipa palm sap. This approach is likely preventing early microbial activity and fermentation without the need of proper packaging, preserving the freshness and quality of the sap more effectively than adding the preservative after collection.

## Introduction

In Sarawak, nipa palm sap is a traditional beverage consumed by the locals, meanwhile nipa palm sugar or *gula apong* is a main ingredient to make some traditional desserts such as *sarang semut*, *kuih ros* and *kuih penyaram*. Fresh nipa sap, which is also locally known as *air neera* or *air sadap*, has a sweet and fruity fragrance, without any presence of higher alcohols and acetic acid content [1]. However, nipa palm sap has a short shelf-life and is prone to fermentation. Due to unhygienic harvesting processes, nipa palm sap remains fresh only for 1–2 h [2]. Nipa palm sap also contains high sucrose which makes it an ideal medium for bacteria under direct sunlight as it contains yeast (*Saccharomyces* sp.) and bacteria (*Acetobacter* sp.) [3–5]. Spoiled sap has an acidic taste of sugar that may hinder the palm sugar production [6]. Hence, the farmers commonly used preservatives to prevent the sap damage. The addition of preservatives helps to slow down natural fermentation, hence increasing the shelf-life of nipa palm sap and producing more derivative products. Several chemicals have been used to control the natural fermentation in palm saps including toluene, cupric sulphate, sulphanilamide, sodium metabisulfite, sorbic acid, benzoic acid and potassium sorbate. Some of these substances are not approved as food additives today [7]. Karseno and Yanto [8] have reported that most farmers in Kebasen district have been using SMS to preserve the nipa palm sap. Another research has reported that shelf-life of nipa palm sap can be extended by 6 months with the use of SMS and pasteurization [9]. Therefore, this study aims to examine the advantage of combining water, sodium metabisulfite (SMS) and potassium sorbate (PS) on the shelf life of nipa palm sap. It focused on how this combination maintains the quality of the sap can before it is processed into palm sugar without the need for proper packaging. A mixture of SMS and PS with water is chosen rather than without water since PS are easily oxidized when exposed to air and SMS alone produces a pungent smell and is difficult to dissolve.

## Methods

### Preparation of aqueous sap preservatives

Neutralizing aqueous solution for high quality or the preservation of nipa palm sap was prepared in the different stoichiometric ratios. Tables 1 and 2 shows the formulation and characteristics of the preservative for each formulation code. Double boiling method with continuous stirring on a hot plate has been applied and water served to ensure both potassium sorbate and sodium metabisulfite mix thoroughly.

**Table 1:** Different concentrations of neutralizing aqueous solution for high quality of Nipa palm sap

Ingredients (g)	Formulation		
	F1	F2	F3
Potassium Sorbate	15.99	27.10	8.79
Sodium Metabisulfite	2.05	3.47	1.13
Water	81.96	69.43	90.08

**Table 2:** Characteristics of neutralizing aqueous solution for high quality of Nipa palm sap

Formulation/ Characteristics	F1	F2	F3
**Physical appearance**	Yellowish	Dark Yellow	Pale Yellow
**pH**	6.94	7.31	6.86
**TDS (ppt)**	10	10	10
**Conductivity**,** EC (µs/cm)**	20	20	20
**Salinity (g/kg)**	0	0	0
**Bulk density (g/mL)**	5.047 g / 4.6 mL = 1.097	5.053 g / 4.4 mL = 1.148	5.099 g / 5 mL = 1.0198
**Tapped density (g/mL)**	1.097	1.148	1.0198
**Hausner’s ratio**	1	1	1
**Preservative Cessation time (s)**	1	1	1

Different palm sap preservative compositions may develop preservatives having different strength on preserving and taste of the sap thereafter. However, in this study, F3 was chosen as it was slightly acidic. Hence, it should be able to inhibit the microbial growth effectively, thereby extending the shelf-life of the nipa palm sap.

### Sample collection

The nipa palm saps used were collected from Kampung Pinggan Jaya, Sarawak. 9 different palms were randomly chosen. Three containers (A1, A2 and A3) had been added with sap preservative (≥ 15 g) before it was used to collect the sap. All of the samples were obtained 12 h after it was tapped in the evening. An additional six samples were obtained, and the preservative was added to samples B1, B2 and B3, followed by gentle shaking. Meanwhile, C1, C2 and C3 act as the experimental control.

### pH determination

The pH of nipa sap was determined using a pH meter after calibration with a standard buffer at room temperature. The pH was measured, and a graph of data was generated based on its average. The data then were analysed using one-way ANOVA. A significant difference exists when the F-count value is greater than the F-table. Differences were considered significant at *p* < 0.05.

### Determination of dissolved sugar, reducing sugars and organic acids

The dissolved sugar content (%), specifically sucrose was determined using a refractometer dissolved sucrose brix meter (on-site) and the average for each group was calculated. Reducing sugars and organic acids content were analysed after 24 h. Reducing sugars were analysed using dinitrosalicylic acid (DNS), while organic acids (lactic and acetic acid) content was analysed using high-performance liquid chromatography (HPLC). 0.005 M of sulfuric acid, H_2_SO_4,_ was used as the mobile phase with 0.8 mL/min flow rate at column temperature of 60 °C as previously described by Jaraee et al. [5].

### Sensory analysis

Sensory analysis was done with a total of four panellists. Each panel was asked to evaluate the sensory characteristics of the sap samples including colour, aroma and taste of sap. One-way ANOVA was used to evaluate the significant difference among the data. Differences were considered significant at *p* < 0.05.

### Determination of microbial growth activity

With a slight modification, the total bacterial count (TBC) was determined according to the method adopted by Jaraee et al. [5]. The microbial growth of untreated and treated nipa sap was determined by using plates agar count. A surface plating using the standard spread plate method was done. 1 mL of treated nipa sap containing preservative was serially diluted with saline solution to prepare some appropriate dilutions (10^− 1^, 10^− 2^, 10^− 3^, 10^− 4^, 10^− 5^ and 10^− 6^). 0.1 mL of each dilution was poured and spread plated on respective agar plates. Each sample was done in triplicate. The agars were incubated at 37 °C for 24 h. After that, the colonies in incubated plates were counted. Another experiment was done by replacing the treated nipa sap with untreated nipa sap.

## Results and discussion

### Effect of preservatives on physical characteristics of nipa palm sap

#### Colour

**Fig. 1:**
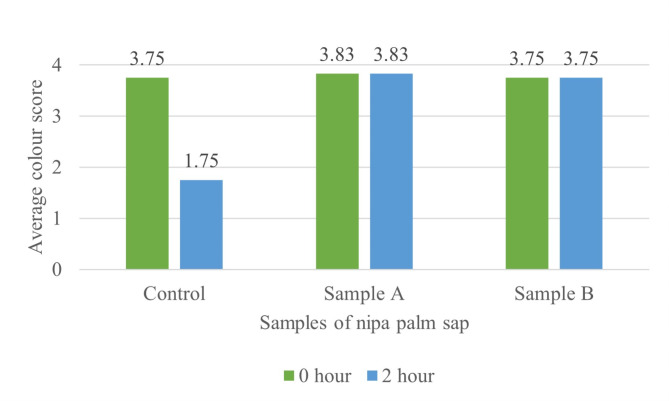
Effect of preservative on the colour of nipa palm sap. (1 = white; 2 = cloudy white; 3 = slightly yellow; 4 = yellow)

Colour of the sap can be used to indicate the sap’s quality during storage before fermentation. According to the local villagers, the yellowish colour of the *Nypa fructicans* sap indicates a good sugar quality and the freshness of the sap. The average colour value of nipa palm sap samples was 3.75 (yellowish); 3.83 (yellowish) and 3.75 (yellowish). No significant differences (*p* > 0.05) were observed on the colour of the nipa palm sap when the samples were first collected. The colour of Sample A was slightly darker than the control, and it still retained its colour after 2 h. For Sample B, the preservative was applied upon the collection. Initially, its physical appearance was the same as the control. After 2 h, its colour remained unchanged. Control sample changed into cloudy white during the 2 h of storage period, indicating that the fermentation took place (Fig. 1). This finding agrees with the results of Azis [10], and Victor and Orsat [11] that a high pH causes the sap of *Nypa fructicans* to appear yellowish and translucent, while a low pH causes the sap to appear cloudy.

#### Aroma

**Fig. 2:**
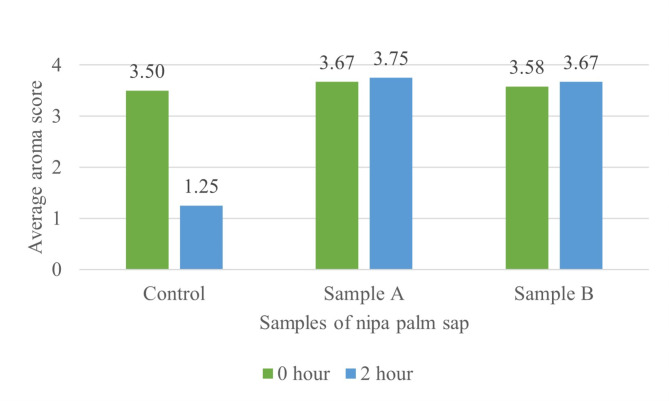
Effect of preservative on the aroma of nipa palm sap. (1 = not strong; 2 = slightly strong; 3 = strong; 4 = very strong)

The results of the analysis showed (Fig.2) that the addition of preservatives had no significant effect (*p* > 0.05) on the aroma of nipa palm sap during the initial storage period (0-hour). The panellists are still able to detect the strong aroma of sap in the treated samples. Initial score of Sample A slightly differs from the control sample and Sample B might be due to its sap concentration. The high concentration of nipa palm sap tends to produce a stronger aroma of the sap [12]. This data also shows that preservatives were able to slow down the natural fermentation in nipa palm sap. In contrast, the average value of nipa palm sap aroma in the control sample, Sample A and Sample B showed a significant effect (*p* < 0.05) after 2 h of storage with score of 1.25 (not strong), 3.75 (strong) and 3.67 (strong), respectively. Aroma is formed by the activities of various enzymes which most of it are secreted in the food matrix by fermenting microbes [13]. For example, the activity of LAB during the fermentation process produces volatile organic compounds that may contribute to the aroma of fermented nipa palm sap [5, 14–16].

#### Taste

**Fig. 3:**
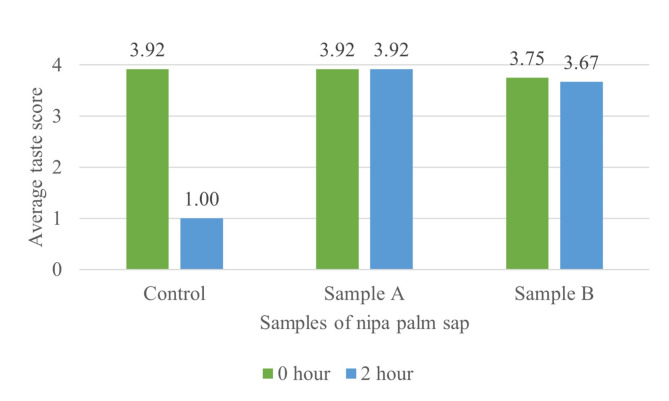
Effect of preservative on the taste of nipa palm sap. (1 = less sweet; 2 = slightly sweet; 3 = sweet; 4 = very sweet)

The results of analysis showed that the use of preservatives had a significant effect (*p* < 0.05) on sweetness of nipa palm sap after 2 h of storage (Fig. 3). This data shows that the preservative used can inhibit microbial growth. Potassium sorbate dissociates into sorbic acid when dissolved in water, hence creating a less favourable environment for microbial growth. Sorbic acid acts as an effective inhibitor of moulds, yeasts, and fungi in which it can be used as antifungal and antimicrobial preservatives in a variety of food, cosmeceutical, and pharmaceutical products [17]. The yeast and bacteria in the nipa palm sap are unable to consume all of the sucrose in the sap, implying that the sweet taste of palm sap remains strong. Over time, the control sample became less sweet or sour which might be due to a higher sugar content that causes the fermentation bacteria to produce higher acidity [4, 5, 18, 19]. This is supported by McFeeters [20] observation that showed taste changes during fermentation occur because of the interaction between active enzymes of the raw material with the metabolic activities of microorganisms.

### Effect of preservatives on chemical characteristics of nipa palm sap

**Fig. 4:**
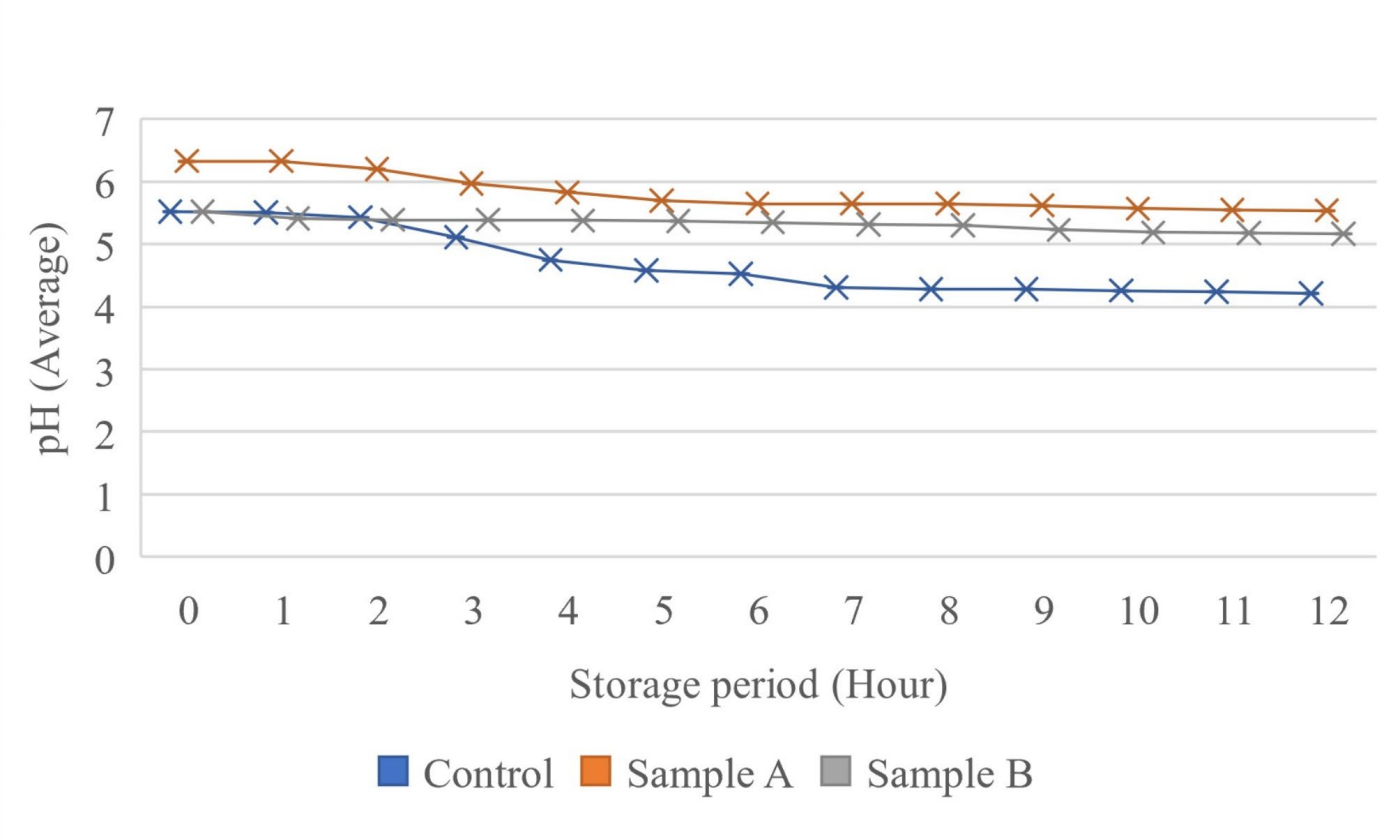
Changes in acidity (pH) of nipa palm sap during 12 h of storage

Throughout the storage, the pH level in all sample groups decreased significantly (*p* < 0.05). In Fig. 4, it was observed that the initial pH Sample A was the highest (6.33 ± 0.17) compared to the control sample and Sample B (both 5.52 ± 0.15). The control and Sample B, collected 12 h after harvesting without preservatives, likely began to ferment. Environmental exposure during sap collection can introduce microbes and contaminants [21]. For Sample A, the preservatives were added before sap collection, inhibiting microbial activity and delaying fermentation [7, 8]. After 1 h, the pH of Sample A remained unchanged, while pH of Sample B dropped to 5.41 ± 0.01 because the preservative was added post-collection, allowing microbial fermentation to proceed more rapidly. The control sample showed a slight pH drop (from 5.52 ± 0.15 to 5.51 ± 0.15) over 1 h. pH drops of Sample B was more noticeable than control sample after 1 h of storage period and it might be due to the rapid shift in the pH as the preservative begins to inhibit microbial activity and stabilize the pH, resulting in the pH maintaining at a more stable level during the remaining storage period. In contrast, microbial activity and fermentation in the control sample became more rapid, which led to a continuous and significant drop (*p* < 0.05) in pH over time. The analysis of variance results (Table 3) showed that the F-count value is greater (33.62585) than the F-Table (3.259446). Thus, the use of preservatives significantly affects (*p* < 0.05) the pH levels and the shelf-life of nipa palm sap.The antimicrobial effects of these preservatives inhibited microbial activity, thus reducing the rate of fermentation. The decrease in pH indicates an increase in the production of lactic and acetic acids during the fermentation of nipa palm sap, consequently leading to an increase in ethanol production [5]. Acid tolerant microbes like acetic acid bacteria (AAB) and lactic acid bacteria (LAB) can remain active despite increased acidity [14].

**Table 3:** Analysis of variance

Source of Variation	SS	df	MS	F	*P*-value	F crit
Between Groups	8.217554	2	4.108777	33.62585	5.8E-09	3.259446
Within Groups	4.398877	36	0.122191			
Total	12.61643	38				

**Fig. 5:**
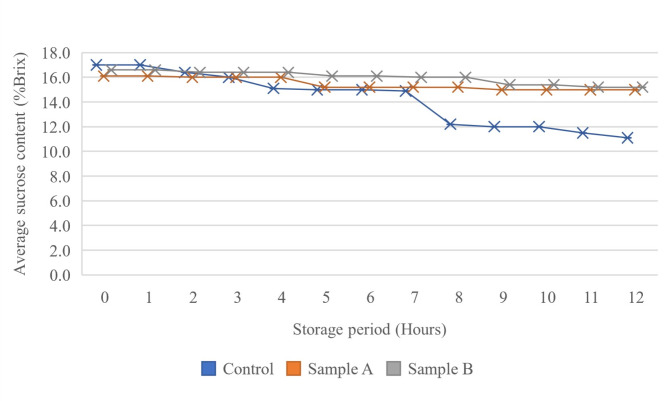
Changes in sucrose content (%Brix) during storage of nipa palm sap

**Fig. 6:**
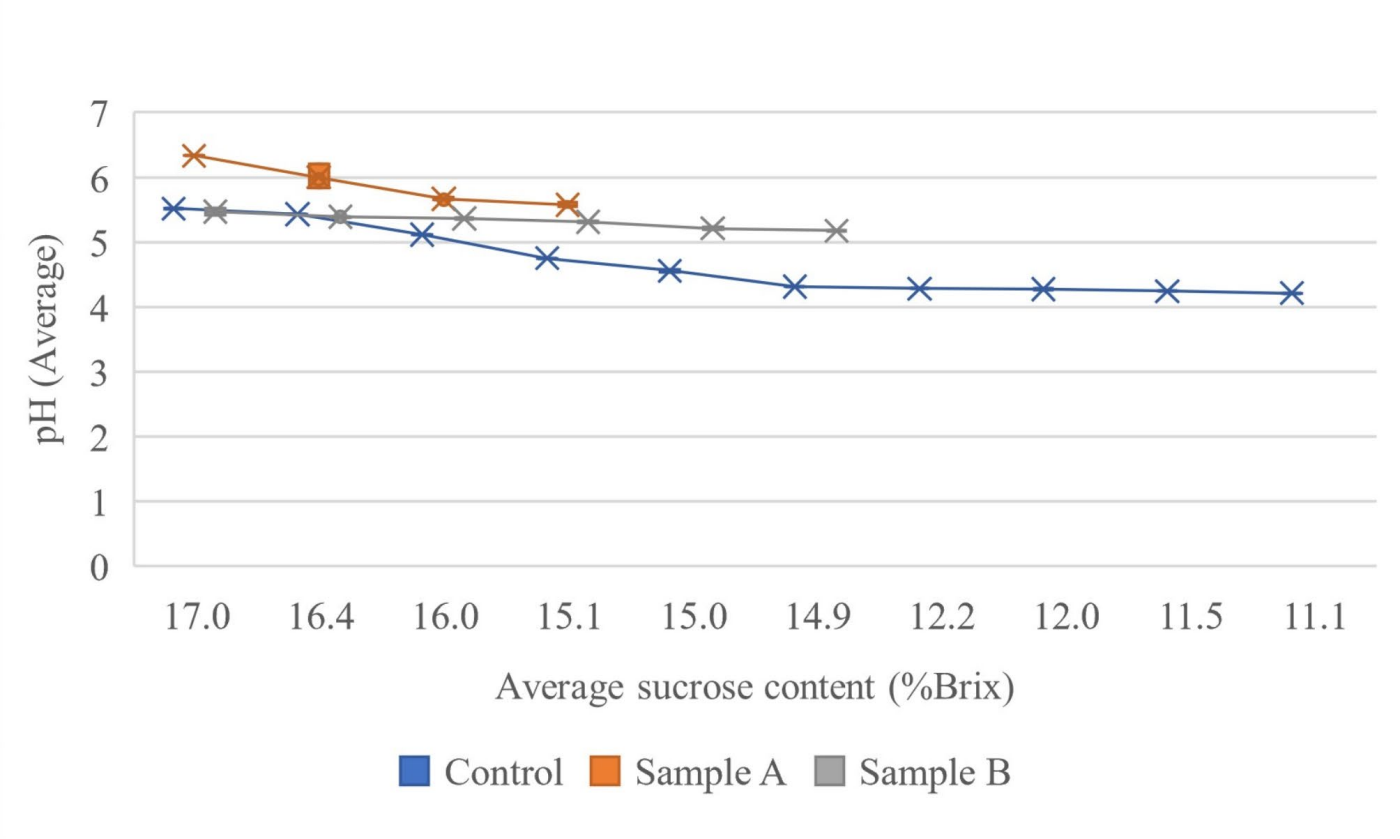
Relationship between pH and sucrose content (%Brix) of nipa palm sap

The sucrose content in all sample groups decreased significantly (*p* < 0.05) indicates that the sap produced different sugar composition throughout the storage period (Fig. 5). The control sample showed a significant drop in sugar concentration from 17.00 ± 0.03 to 11.1 ± 0.03% Brix, correlating with a pH drop from 5.52 ± 0.15 to 4.21 ± 0.01 (Fig. 6). This decline may result from microbial contamination during tapping, causing sucrose inversion due to acidic conditions. Sample A and B maintained higher sugar levels, indicating that preservatives slowed fermentation and reduced acidity, effectively preserving sweetness and extending the shelf-life of nipa palm sap [22]. Sample B had a higher initial sugar level than Sample A, possibly due to differences in sap composition. Towards the end of the storage, sugar levels in Sample A and Sample B dropped from 16.10 ± 0.03 to 15.00 ± 0.03% Brix and 16.60 ± 0.03 to 15.20 ± 0.03% Brix. SMS and PS helps stabilize the sugar which minimizes or prevents the sugar inversion that can occur due to the acidic conditions [23]. The findings align with Jaraee et al. [5], indicating an increase in reducing sugar. The chemical composition of nipa palm sap samples after 24 h of storage is shown in Table 4. It was clearly shown that the initial enzymatic breakdown of sucrose into glucose and fructose may have been slowed down by the preservatives, resulting in lower concentrations of reducing sugars. The sugar composition of nipa palm sap correlated with the studies in Indonesia [24], Thailand [25], Peninsular Malaysia [26] and East Malaysia [5]. However, the sugar composition of nipa palm sap in this study was lower than Jaraee et al. [5] but higher than that in the other studies. This is because sugar composition of nipa palm could differ from place to place, influenced by the tapping process and environmental condition [26]. Lactic acid and acetic acid were also highest in the control sample due to ongoing microbial activity which ferment the sugars in nipa palm sap over time. The preservatives in the treated samples help maintain lower levels of these acids in the nipa palm sap.

**Table 4:** The chemical composition of fresh nipa palm sap after 24 h of storage

Parameters	Control*	Sample A*	Sample B*
**pH**	4.21 ± 0.01	5.48 ± 0.01	5.10 ± 0.01
**Total sugar (g/L)**	172.8 ± 1.6	200.8 ± 1.2	201.3 ± 0.8
**Sucrose (g/L)**	108.0 ± 0.03	149.0 ± 0.03	150.0 ± 0.03
**Glucose (g/L)**	26.7 ± 1.8	19.7 ± 0.8	19.5 ± 1.0
**Fructose (g/L)**	38.1 ± 1.0	32.1 ± 1.2	31.8 ± 0.5
**Lactic Acid (g/L)**	1.10 ± 0.01	0.93 ± 0.01	0.97 ± 0.02
**Acetic Acid (g/L)**	0.09 ± 0.02	0.05 ± 0.01	0.07 ± 0.01

*Values are given as average ± error from triplicate measurements

### Effect of preservatives on microbial activity of Nipa palm sap

**Table 5:** Average of Total Bacterial Count (CFU/mL) of nipa palm sap at different concentrations

Sample/ Dilution	Average of Total Bacterial Count (CFU/mL)					
	10^− 1^	10^− 2^	10^− 3^	10^− 4^	10^− 5^	10^− 6^
Control	TNTC	TNTC	2.83 (± 0.02) x 10^5^	1.89 (± 0.05) x 10^6^	1.51 (± 0.06) x 10^7^	1.30 (± 0.09) x 10^8^
Sample A	1.56 (± 0.22) x 10^3^	1.18 (± 0.12) x 10^4^	1.03 (± 0.08) x 10^5^	TFTC	TFTC	TFTC
Sample B	TNTC	1.30 (± 0.14) x 10^4^	1.14 (± 0.13) x 10^5^	0.83 (± 0.13) x 10^6^	0.40 (± 0.04) x 10^7^	TFTC

(TNTC = Too numerous to count; TFTC = Too few to count)

TBC was done to determine the microbial quality of nipa palm sap after 24 h of storage. The microbial growth of the nipa palm sap was analysed after 24 h of incubation as shown in Table 5. Results obtained showed that the preservative, composed of PS and SMS, works effectively against bacteria. It acts as a protective agent, preventing the spoilage and deterioration of the sap during storage. In the formulation, water was used to ensure PS and SMS dissolve and mix properly, but it also aids in converting PS to sorbic acid. Sorbic acid possesses antifungal and antimicrobial properties. Therefore, the combination of SMS and sorbic acid kills or inhibits growth of microbes responsible for spoiling nipa palm sap [27]. A higher number of TBC was observed in the control sample (> 250 colonies) as it does not have any substances that impede the growth and proliferation of bacteria which naturally occur in the nipa palm sap. Therefore, as the control sample lacked microbial inhibitory measures, its TBC was higher than that of sample A and B, which contained a preservative. The control sample was susceptible to microbial contamination during collection and storage period without the presence of preservative. According to Oluwole et al. [15], nipa palm sap is rich in nutrients and serves as an ideal medium for the growth of bacteria. The natural sugars and other components in the sap provide a favourable condition for microbial colonization and reproduction [5, 19, 20]. Both sample A and sample B contain preservatives, however the number of TBC in sample A was lower than sample B. The time of the application of preservative influencing the microbial proliferation in the nipa palm sap. In sample A, the preservative was introduced before starting the tapping process. This approach most likely causes the microbial growth to be inhibited at an early stage and any contaminants that might have occurred during the collection process are immediately encountered by the antimicrobial agents. Conversely, in Sample B, the preservative was added after the nipa palm sap was collected. The gap in time allows the possible contamination and growth of bacteria during the collection process, resulting in higher number of TBC in sample B compared to sample A. From the results, it was clearly shown that applying preservative prior to the tapping process effectively minimizes the risk of microbial growth.

### Limitations

Different palm sap compositions aforesaid may develop preservatives having different strength on preserving and taste of the sap. Hence, the main limitation of this study is the sap composition, influenced by factors such as tree age, location and environmental conditions, making it challenging to standardize the experiments. Temperature, humidity, and exposure to light can impact microbial growth rates and enzymatic activities, thereby affecting the preservation process. The use of SMS and PS had relatively no effect on colour, aroma and taste of nipa palm sap as the nature of these materials were odourless, but it had limitations. Excess concentration of these preservatives could cause a bitter taste. Additionally, the study lacks data on soil salinity which can significantly impact the sap quality. Further studies should include soil salinity measurement to provide a more comprehensive analysis.

## Data Availability

No datasets were generated or analysed during the current study.
